# Misophonia in Singaporean Psychiatric Patients: A Cross-Sectional Study

**DOI:** 10.3390/ijerph15071410

**Published:** 2018-07-04

**Authors:** Tian Ci Quek, Cyrus SH. Ho, Carol C. Choo, Long H. Nguyen, Bach X. Tran, Roger C. Ho

**Affiliations:** 1 Department of Psychological Medicine, Yong Loo Lin School of Medicine, National University of Singapore, Singapore 119007, Singapore; pcmrhcm@nus.edu.sg; 2 Department of Psychological Medicine, National University Health System, Singapore 119228, Singapore; su_hui_ho@nuhs.edu.sg; 3 College of Healthcare Sciences, James Cook University, Singapore 387380, Singapore; carol.choo@jcu.edu.au; 4 Institute for Global Health Innovations, Duy Tan University, Da Nang 550000, Vietnam; long.ighi@gmail.com; 5 Institute for Preventive Medicine and Public Health, Hanoi Medical University, Hanoi 100000, Vietnam; bach.ipmph@gmail.com; 6 Department of Health, Behavior and Society, Johns Hopkins Bloomberg School of Public Health, Baltimore, MD 21205, USA; 7 Vietnam Young Physicians’ Association, Hanoi 100000, Vietnam

**Keywords:** misophonia, selective sound sensitivity syndrome, sound sensitivity, anxiety, depression

## Abstract

Misophonia, also known as selective sound sensitivity syndrome, is a condition characterized by strong dislike of specific sounds with accompanying distressing reactions. To date, misophonia is still poorly understood. This study aimed to identify factors associated with severity of misophonic symptoms in Singaporean psychiatric patients. Ninety-two psychiatric patients were recruited from a large teaching hospital in Singapore in a cross-sectional study. Socio-demographics, severity of depression, anxiety and stress, and severity of misophonic symptoms were analyzed. Correlation analysis showed that anxiety, depression, and stress scores—as measured by the Depression, Anxiety and Stress Scales-21 (DASS-21)—were significantly positively correlated with the Amsterdam Misophonia Scale (A-MISO-S) scores. After adjustment for confounding factors, multivariate regression analysis showed that anxiety (β = 0.385, *p* = 0.029) remained significantly associated with A-MISO-S. Age, gender, depression, and stress were not significantly associated with the severity of misophonia. The findings showed that the severity of anxiety was associated with severity of misophonia in Singaporean psychiatric patients. Further research is needed to explore the nature of misophonia and its relationship with other psychiatric disorders.

## 1. Introduction

Misophonia—literally “hatred of sound” and also known as selective sound sensitivity syndrome—is characterized by an intense dislike of specific sounds and is accompanied by distressing emotional and physical reactions [1,2]. Recently, this condition has been expanded to include sights and images sufferers see that can provoke similar reactions [3]. These stimuli are termed “triggers” by sufferers and are highly specific. Examples of auditory triggers described include chewing, low frequency bass sounds, pen clicking, and typing [2], while visual triggers described include pointing of fingers, legs jiggling, and the twirling of hair [4]. After an in-depth study into triggers and reactions of 26 misophonics by Dozier and Morrison [3], the authors concluded that the severity of misophonic reactions are correlated with the amplitude and length of the trigger stimuli. A controlled study by Edelstein et al. [2] demonstrated that misophonics increases skin conductance responses in response to auditory triggers, leading to the conclusion that a heightened autonomic response to triggers may be present in sufferers.

Studies into the nature of misophonia have been inconclusive [5], although it is evident that misophonia causes major functional impairment in sufferers [6]. Given the prevalence of trigger sounds, which is inevitable in modern society, and the rising number of self-identified misophonia sufferers in online support groups, it is imperative that this condition garner more attention among researchers and clinicians alike.

Misophonia has been described in the literature possibly as part of a psychiatric disorder [6] or as a separate entity with its own set of psychopathology [7,8]. A recent large-scale online study of more than 300 misophonia sufferers conducted by Rouw and Erfanian [9] found post-traumatic stress disorder (PTSD) to be associated with the severity of misophonia. A study in Amsterdam of 42 self-reported misophonics conducted by Schröder et al. in 2013 [1] found 52.4% of sufferers had comorbid obsessive-compulsive personality disorder (OCPD). A recent large-scale study of 483 undergraduates conducted by Wu et al. [6] in 2014 found that anxiety and depressive symptoms were correlated with misophonia symptoms. However, it remains unclear if studies done in other countries can be extrapolated to the local Singaporean context. To expand on this finding, the aim of this study was to investigate factors associated with the severity of misophonia in psychiatric patients in Singapore.

## 2. Materials and Methods

The methodology adopted in this study is based on a previous cross-sectional study conducted by Ho et al. [10].

### 2.1. Recruitment of Patients

The study was approved by the Domain Specific Review Board (DSRB) of National University Hospital (reference number: 2016/01377; approval period: 14 February 2017 to 13 February 2018). Patients were recruited from the outpatient psychiatric clinic at the National University Hospital located in Singapore from 14 February 2017 to 13 February 2018. Patients over the age of 21 were recruited. These patients were not preselected based on whether they self-reported misophonic symptoms. The exclusion criteria of this study were patients with severe cognitive deficits (e.g., learning disability and dementia) and those showing evidence of alcohol and/or drug abuse. All patients recruited gave written informed consent before their participation in the study.

### 2.2. Demographics and Data Collection

Socio-demographic and clinical data were obtained from patients’ medical records and clinical interviews with the research assistant. This included age, gender, ethnicity, marital status, and primary diagnosis at time of interview.

### 2.3. Assessment of Psychiatric Status

Psychiatric evaluation was assessed with the self-rated Depression, Anxiety, Stress Scales 21 (DASS-21) in the presence of a research assistant during the clinical interview. The DASS-21 consisted of three 7-items, self-report subscales that quantified the severity of the patients’ depression, anxiety, and stress over the past week. The DASS-21 was validated to reliably assess these three dimensions in clinical and nonclinical groups [11].

### 2.4. Assessment of Misophonia Severity

The severity of the patients’ misophonia was assessed with the self-rated Amsterdam Misophonia Scale (A-MISO-S), which was developed by Schroder et al. based on the Yale–Brown Obsessive Compulsive Scale. It was developed after the authors studied 42 patients who reported they had misophonia [1]. To date, the scale has not been validated.

Before completing the A-MISO-S, the research assistant educated the patients on what misophonia is and gave examples of the emotional reactions (e.g., anger, fear) and physical reactions (e.g., palpitations, muscle tension) sufferers might experience [3].

### 2.5. Statistical Analysis

All statistical analyses were performed using the SPSS statistical package program version 24.0 (SPSS Inc., Chicago, IL, USA). Unless otherwise specified, values were expressed as mean ± SD. Correlations among the continuous variables were studied using Pearson’s correlation coefficient (r). When the data did not conform to a normal distribution, non-parametric Spearman’s rank correlation test was used. Using the A-MISO-S score as the dependent variable, univariate and multivariate linear regressions were performed to identify factors associated with severity of misophonia. The validity of the regression model was ascertained by a tolerance test and analyses of residuals. The tolerance test was adopted to detect multicollinearity of the confounding independent variables and a value of >0.6 was accepted. Statistical significance was set at *p* < 0.05.

## 3. Results

### 3.1. Demographics

The demographic features of the patients are illustrated in Table 1. Ninety-eight patients were invited, and 92 of them gave their consent to participate (93.9% response rate). The mean age was 39.85 ± 13.26 years. The majority of the patients were Chinese (82.6%). At the time of the interview, the most prevalent primary diagnoses of the patients were depression (52.2%), schizophrenia, (22.2%) and anxiety disorders (12.2%).

### 3.2. Correlation among Demographic, Clinical, and A-MISO-S Variables

The correlation coefficients among demographic, clinical, and A-MISO-S characteristics are illustrated in Table 2.

A-MISO-S score was negatively related to age (r = −0.23, *p* = 0.026). A-MISO-S score was also significantly positively correlated to depression (r = 0.47, *p* < 0.001), anxiety (r = 0.56, *p* < 0.001), and stress (r = 0.50, *p* < 0.001).

Stress was significantly positively correlated to depression (r = 0.75, *p* < 0.001) and anxiety (r = 0.83, *p* < 0.001). Anxiety was significantly positively associated with depression (r = 0.73, *p* < 0.001).

### 3.3. Factors Associated with A-MISO-S in Patients

Using A-MISO-S as the dependent variable (Table 3), univariate regression indicated that depression (*p* < 0.001), anxiety (*p* < 0.001), and stress (*p* < 0.001) were positively associated with severity of misophonia. Gender was also significantly associated with the severity of misophonia. After adjustment for confounding factors in multivariate regression, only anxiety (β = 0.385, *p* = 0.029) remained significantly associated with A-MISO-S.

### 3.4. Types of Sound

The identifiable theme of trigger sounds reported by patients was that these sounds were produced by someone else other than themselves. The trigger sounds reported by patients included chewing, footsteps sound from neighbors, low-frequency bass sound, and specific people talking, especially in a high-pitched tone. When asked by the research assistant if they would experience misophonic symptoms if they had produced such sounds, almost all answered they would not. Some patients who had high A-MISO-S score reported baby crying as their triggers.

## 4. Discussion

### 4.1. Misophonic Symptoms in Anxiety Disorders

Our result showed that the severity of anxiety in psychiatric patients was associated with the severity of misophonia symptoms. This is consistent with Wu et al.’s [6] 2014 study which found that misophonia is correlated with anxiety and that anxiety is a significant mediator between misophonia symptoms and rage. The pathology of anxiety may mean that sufferers become more sensitive and hyper-aware to their environment due to hypothesized dysfunction of the locus coerulus-noradrenaline-sympathetic nervous system [12]. Hence, it could be possible that misophonia is a consequence of their state of anxiety. The challenge for clinicians then would be to determine whether the misophonic symptoms are on a background of a patient’s underlying anxiety disorder or a standalone occurrence with no underlying psychiatric comorbidity. 

Misophonia symptoms also co-occur in patients with other psychiatric disorders, such as OCPD and mood disorders, as found by Schroder et al. [1]. However, this study was limited by a small sample size and the lack of inter-rater reliability for psychiatric diagnostic assessment [5]. Hence, further research should explore the relationship between these psychiatric disorders and misophonic symptoms.

### 4.2. Factors Not Associated with Severity of Misophonia

After adjusting for confounding factors, our results showed that gender and age were not significantly associated with the severity of misophonia symptoms. This is consistent with Wu et al.’s [6] 2014 study of 483 undergraduate students which found no significant gender and age differences in severity of misophonia symptoms.

We found that A-MISO-S was significantly positively correlated with the DASS-21 depression score, a finding comparable to Wu et al.’s study [6]. However, after adjusting for confounding factors, multivariate regression revealed that depression as measured by the DASS-21 depression score was no longer significantly associated with the severity of misophonia symptoms. This could suggest that depression may not play a role in the psychopathology of misophonia.

We also found that A-MISO-S score was not significantly associated with the DASS-21 stress score after adjusting for confounding factors. This could be because the DASS-21 stress scale assesses “features of both anxiety and depression” [11] and hence, the qualities of a patient’s anxiety and depression will be compounded in the stress scale.

### 4.3. Strengths and Limitations

Our study had two main strengths. Firstly, the subjects were recruited from a psychiatric outpatient clinic and hence, the study sample should be representative of psychiatric outpatients in a large hospital in Singapore. Secondly, the DASS-21 is a brief and validated [11] instrument, which allowed us to reliably assess the current psychiatric status of patients during the point of interview. This is a better measure than the primary diagnoses of patients, which may not be the most accurate representation of mental health as the conditions can be well-controlled under the management plan prescribed by psychiatrists.

Nevertheless, our study also had several limitations. Firstly, the DASS-21 is indicative of the severity of depression, anxiety, and stress symptoms based on the subjects’ self-reports, instead of a more objective and comprehensive clinical interview. Secondly, there was no control (non-psychiatric) group for the comparison of DASS-21 scores and A-MISO-S scores. The recent large-scale study of over 300 misophonia sufferers conducted by Rouw and Erfanian [9] found that half of the misophonia sufferers reported no clinical comorbidities. Hence, it could be possible that our study was biased in selecting for the subgroup of misophonia sufferers with existing comorbidities. Thirdly, other factors such as obsessive-compulsive symptoms [6] have also been associated with misophonia, but these factors were not investigated in this study. Fourthly, to date, A-MISO-S has not been validated in studies for assessing the severity of misophonia. Lastly, as this was a cross-sectional study, the causality between anxiety and misophonia could not be ascertained. Future research should examine this relationship in a longitudinal study. Within the limits of this study, we explored factors that were associated with misophonia in Singaporean psychiatric patients and we hope this can prompt further research in misophonia in Asian populations.

## 5. Conclusions

In conclusion, the severity of anxiety in Singapore psychiatric patients was found to be associated with the severity of misophonic symptoms they experienced. This might offer valuable insights into the psychopathology of misophonia, either as part of a psychiatric disorder or as a yet to be understood condition. Further research is needed to explore its relationship and association with other psychiatric disorders, while considering other major systems where the pathology of misophonia could lie.

## Figures and Tables

**Table 1:** Demographic features of patients.

Feature	Mean ± SD (%) N = 92
Age	39.85 ± 13.26
Sex (Female)	52 (56.5)
Ethnicity	
Chinese	76 (82.6)
Malay	4 (4.3)
Indian	11 (12.0)
Others	1 (1.1)
Married	28 (30.8)
Primary diagnosis	
Depression	47 (52.2)
Schizophrenia	20 (22.2)
Anxiety disorders	11 (12.2)
Borderline personality disorder	8 (8.9)
Bipolar disorder	3 (3.3)
Attention Deficit Hyperactivity Disorder	1 (1.1)
Unknown	2 (2.2)
DASS-21 score	
Depression	18.61 ± 11.83
Anxiety	18.68 ± 11.22
Stress	19.93 ± 11.25
A-MISO-S score	7.60 ± 5.62

**Table 2:** Correlation among demographic, clinical, and A-MISO-S variables in patients.

	Age	DASS-21 Depression Score	DASS-21 Anxiety Score	DASS-21 Stress Score	A-MISO-S Score
Age	1.00				
DASS-21 Depression Score	−0.03				
DASS-21 Anxiety Score	−0.15	0.73 **			
DASS-21 Stress Score	−0.03	0.75 **	0.83 **		
A-MISO-S Score	−0.23 *	0.47 **	0.56 **	0.50 **	1.00

* *p* < 0.05 (two-tailed). ** *p* < 0.01 (two-tailed). DASS-21: Depression, Anxiety, and Stress Scales-21; A-MISO-S: Amsterdam Misophonia Scale.

**Table 3:** Univariate and multivariate association between socio-demographics, clinical factors and A-MISO-S.

Variables	Univariate Regression						Multivariate Regression					
	B	SE	β	Adjusted r^2^	F-change	*p*	B	SE	β	Adjusted r^2^	F-change	*p*
**Socio-Demographic Characteristics**												
Age	−0.083	0.044	−0.195	0.027	3.544	0.063	−0.067	0.047	−0.162	0.316	6.352	0.159
Gender	2.340	1.163	0.207	0.032	4.047	0.047 *	0.857	1.059	0.077			0.421
**Clinical Features**												
DASS-21 depression score	0.222	0.044	0.473	0.215	25.075	<0.001 **	0.042	0.068	0.090			0.541
DASS-21 anxiety score	0.295	0.045	0.588	0.338	43.945	<0.001 **	0.192	0.086	0.385			0.029 *
DASS-21 stress score	0.258	0.045	0.522	0.264	32.143	<0.001 **	0.044	0.084	0.089			0.607

* *p* < 0.05 (two-tailed). ** *p* < 0.01 (two-tailed).
